# Adolescent Attachment Profiles Are Associated With Mental Health and Risk-Taking Behavior

**DOI:** 10.3389/fpsyg.2021.761864

**Published:** 2021-12-02

**Authors:** Marjo Flykt, Mervi Vänskä, Raija-Leena Punamäki, Lotta Heikkilä, Aila Tiitinen, Piia Poikkeus, Jallu Lindblom

**Affiliations:** ^1^Faculty of Medicine, Department of Psychology and Logopedics, University of Helsinki, Helsinki, Finland; ^2^Faculty of Social Sciences (Psychology), Tampere University, Tampere, Finland; ^3^Faculty of Medicine, Department of Obstetrics and Gynecology, University of Helsinki, Helsinki, Finland; ^4^Department of Obstetrics and Gynecology, Helsinki University Hospital, Helsinki, Finland; ^5^Department of Clinical Medicine, University of Turku, Turku, Finland

**Keywords:** attachment, latent profile analysis (LPA), adolescence, mental health, risk-taking, substance use, sexual risk

## Abstract

This person-oriented study aimed to identify adolescents’ hierarchical attachment profiles with parents and peers, and to analyze associations between the profiles and adolescent psychosocial adjustment. Participants were 449 Finnish 17–19-year-olds reporting their attachments to mother, father, best friend, and romantic partner and details on mental health (internalizing symptoms, inattention/hyperactivity, and anger control problems) and risk-taking behavior (substance use and sexual risk-taking). Attachment was measured with Experiences in Close Relationships – Relationship Structures (ECR-RS); internalizing, inattention/hyperactivity, and anger control problems with Self-Report of Personality — Adolescent (SRP—A) of the Behavior Assessment System for Children, third edition (BASC-3); substance use with the Consumption scale of the Alcohol Use Disorders Identification Test (AUDIT-C) and items from the Finnish School Health Promotion Study; and sexual risk-taking behavior with the Cognitive Appraisal of Risky Events (CARE). Latent profile analysis identified five attachment profiles: “All secure” (39%), “All insecure” (11%), “Parents insecure – Peers secure” (21%), “Parents secure – Friend insecure” (10%), and “Parents secure – Partner insecure” (19%). “All insecure” adolescents showed the highest and “All secure” adolescents the lowest levels of mental health problems and substance use. Further, parental attachment security seemed to specifically prevent substance use and anger control problems, while peer attachment security prevented internalizing problems. Our findings help both understand the organization of attachment hierarchies in adolescence and refine the role of specific attachment relationships in psychosocial adjustment, which can be important for clinical interventions in adolescence.

## Introduction

Adolescence is a major transitional period in socioemotional, neurocognitive, and behavioral development (Crone and Dahl, 2012). Adolescents extend their attachment bonds beyond parents to encompass friends and intimate partners, thus forming multiple hierarchically organized attachment relationships (Bowlby, 1969; Ainsworth, 1989). Two aspects are important in understanding the hierarchical nature of adolescent attachment: First, analogously to young children (Bowlby, 1969), adolescents often perceive one of the multiple attachment figures as *primary* (Freeman and Simons, 2018), and primary attachments, often to the mother, have been found to be crucial for child and adolescent well-being and socioemotional development (Groh et al., 2017). Second, the multiple attachment bonds in adolescence can differ in their degree of security and closeness (Bartholomew and Horowitz, 1991; Fraley, 2019) and are characterized by three dimensions: *Attachment-related anxiety* indicates a heightened need for closeness to and fear of losing one’s attachment figure. *Attachment-related avoidance* is a tendency to cherish autonomy at the expense of intimacy and keep emotional distance to others. Finally, *secure attachment* involves low levels of both attachment-related anxiety and avoidance, allowing access to both optimal emotional intimacy and autonomy.

Research is available on timing in the reorganization of multiple attachment hierarchies across adolescence (Markiewicz et al., 2006; Rosenthal and Kobak, 2010) and on profiles (or typologies) of attachment relations with multiple figures (Wang and Wang, 2012; He et al., 2018) (see also Supplementary Table 2 for a literature review). However, most studies have applied a narrow conceptualization of multiple attachments, typically focusing only on the balance between parental and peer attachment, but not differentiating the specific roles of mothers and fathers or friends and romantic partners. Mothers and fathers may nonetheless play unique roles in adolescent attachment hierarchies, and romantic relationships become especially salient for development in late adolescence and young adulthood (Collins, 2003; Freeman and Almond, 2010). Both the primacy of the attachment figure and the degree of security or insecurity in multiple attachment relations are vital for adolescents’ mental health (Allen et al., 1996; Wilkinson, 2010) and risk-taking behavior (McKay, 2015; Sentino et al., 2018). This study aims to identify hierarchical attachment profiles in late adolescence (17−19 years) that reflect the degree of attachment anxiety, avoidance, and security towards the mother, father, best friend, and romantic partner. We further examine how the attachment profiles identified using latent profile analysis are associated with adolescents’ mental health and risk-taking behavior.

### Attachment Hierarchies and Profiles in Adolescence

In normative development, peers gradually begin to replace parents in fulfilling adolescents’ attachment needs and may sometimes become their primary attachment figures by late adolescence (Kobak et al., 2007). Empirical studies confirm that adolescents decrease the use of both parents in fulfilling their attachment needs from middle (13−15 years) to late adolescence, yet mothers continue to be a stable source of attachment security in late adolescence (Nickerson and Nagle, 2005; Markiewicz et al., 2006; Rosenthal and Kobak, 2010; Viejo et al., 2018).

Research further shows that with increasing age, adolescents’ attachment hierarchies show more variability and higher fluidity with specific attachment figures (Friedlmeier and Granqvist, 2006), and by young adulthood (19−25 years) attachment hierarchies become less differentiated (Rosenthal and Kobak, 2010). Especially romantic partners seem to rise in attachment hierarchy by late adolescence or young adulthood to provide support and affectional bonds (Rosenthal and Kobak, 2010). However, attachment to a romantic partner has been shown to replace attachment to friends rather than attachment to parents (Umemura et al., 2018a).

The quality of attachment may also affect the timing of attachment hierarchy reorganization from parents to peers. In particular, insecurity in relationship with parents may urge adolescents to rely early on peers due to a lack of support and bonding with parents (Weiss, 1991; Freeman and Brown, 2001; Kobak et al., 2007). In line with this view, research shows that insecure-anxious and insecure-avoidant attachment relationships are associated with premature attachment hierarchy reorganization in adolescence (Mayseless, 2004; Friedlmeier and Granqvist, 2006; Pitman and Scharfe, 2010). Some research suggests that specifically insecure attachment to mother, but not to father, is associated with using peers as primary attachment figures (Friedlmeier and Granqvist, 2006; Markiewicz et al., 2006; Pitman and Scharfe, 2010). Nevertheless, the results are not univocal, as for example in a study by Umemura et al. (2018a) the timing of reorganization in adolescents’ attachment hierarchies was independent of whether relationships were secure or insecure.

The person-oriented approach (Magnusson, 1999; Bergman and Lundh, 2015) offers insightful ways to capture the diversity of multiple hierarchically organized attachment relationships in adolescence. The paradigm stresses unique individual experiences, and aims to statistically identify distinct profiles, that is, homogeneous subgroups of individuals based on patterns across multiple variables (Bergman and Trost, 2006). This stands in contrast to the more common variable-oriented approach that typically assumes the independence of variables and tends to focus on the whole-sample level effects. Concerning attachment, the person-oriented approach allows the possibility to identify naturally occurring profiles based on both the relationship-specificity and the degree of anxiety, avoidance, and security in attachment bonds, resulting in empirical representation of hierarchical attachment organizations.

To our knowledge, no previous studies in Western cultures have applied a person-oriented approach to adolescent attachment profiles. However, two Asian studies have used latent profile analysis (LPA) to identify attachment profiles based on multiple relationships (Wang and Wang, 2012; He et al., 2018). Regarding these studies, in a sample of nearly 1000 Chinese adolescents (M_age_ 15.9 years), He et al. (2018) identified four attachment profiles of parents and peers: “All secure” (27.2%) referring to secure attachment and “All insecure” (21.4%) referring to insecure attachment to both parents and peers, as well as “Parents secure – Peers insecure” (32.3%) and “Parents insecure – Peers secure” (19.1%) profiles. The study did not differentiate between attachment to a best friend and a romantic partner, nor between mother and father. By contrast, Wang and Wang (2012) assessed specific attachment profiles of best friend and romantic partner, in addition to mother and father, among 302 Chinese college students. They also identified “All secure” (66.3%) and “Parents insecure – Peers (=friend and romantic partner) secure” (7.7%) profiles. However, their two additional profiles differed from He et al. (2018) in revealing “Parents and friend secure – Romantic partner insecure” (7.7%) and “All average secure” (18.2%) profiles.

Other, variable-oriented studies have divided adolescents’ multiple attachment relationships into groups based on the means and standard deviations of two dimensions: security to parents and security to peers (Raja et al., 1992; Laible et al., 2000; Laghi et al., 2009). These studies have formed groups comparable to profiles identified by He et al. (2018), yet with different distributions. Most adolescents belonged to either the “All secure” (Raja et al., 1992; Laghi et al., 2009) or the “Parents secure – Peers insecure” (Laible et al., 2000) groups. The groups reflecting insecure parental attachment were the smallest in size, with adolescents belonging to either the “All insecure” (Raja et al., 1992; Laible et al., 2000) or “Parents insecure – Peers secure” (Laghi et al., 2009) groups. It is important to note, however, that using pre-defined cut-off points to form attachment groups is arbitrary and can lead to unrealistic estimates of their distribution. To conclude, research is lacking on adolescents’ naturally occurring attachment organizations involving relationships to mothers, fathers, best friends, and romantic partners. Further, it is unclear whether adolescents’ hierarchical attachment profiles are similar in different cultures.

### Attachment Hierarchies and Adolescent Psychosocial Adjustment

Research has analyzed how attachment hierarchies are associated with adolescent psychosocial adjustment, including mental health problems, risk-taking behavior, and positive outcomes such as prosocial behavior. Both person-oriented profile studies (Wang and Wang, 2012; He et al., 2018) and variable-oriented group-based studies (Raja et al., 1992; Laghi et al., 2009) indicate that adolescents with “All secure” attachments show the best psychosocial adjustment, while those with “All insecure” attachments show the poorest adjustment, indicated by high depression, anxiety, and aggression symptoms. We refer to these tenets as *security-resilience* and *insecurity-vulnerability hypotheses*. It is plausible that multiple secure relationships offer the best (actual and perceived) psychosocial support for adolescents to meet the different challenges in their lives, important for well-being. In contrast, complete lack of access to secure attachment bonds is likely to be detrimental for mental health, involving poor regulatory functioning and maladaptive coping.

Concerning the relative contributions of parental and peer attachment for adolescent psychosocial adjustment, two different hypotheses can be presented: *parental primacy hypothesis* and *peer compensation hypothesis*. According to the *parental primacy hypothesis*, adolescents who must compensate their insecure and unsupportive parental relationships by seeking early affiliation and safety from peers are at heightened risk for mental health, social, and behavioral problems (Markiewicz et al., 2006; Kobak et al., 2007). However, the *peer compensatory hypothesis* suggests that secure attachment to peers when parental attachment is insecure can potentially protect adolescents against some problems (Laible et al., 2000; Mayseless, 2004; He et al., 2018).

In support of the *parental primacy hypothesis*, research shows that insecure attachment to parents when combined with reliance on peers may be harmful for adolescent psychosocial adjustment. First, the person-oriented study by He et al. (2018) found that adolescents in the “Parents insecure – Peers secure” profile showed higher levels of depression, anxiety, and stress symptoms than those in the “Parents secure – Peers insecure” profile. Second, variable-oriented studies have reported that adolescents who prefer peers to parents as attachment figures show a high level of negative affectivity (Umemura et al., 2018b) and internalizing and externalizing symptoms (Rosenthal and Kobak, 2010). Similarly, insecure attachment to parents combined with secure attachment to peers was found to be associated with adolescent depression and stress (Oldfield et al., 2016). Third, variable-oriented studies also show that adolescents who rely on peers for attachment support are more likely to show aggression (Oldfield et al., 2016, 2018), affiliate with deviant peers (Abbott et al., 2019), and have higher levels of problematic alcohol use (McKay, 2015). Thus, we expect that adolescents with the “Parents insecure – Peers secure” profile are at higher risk for both mental health problems and risk-taking behavior than adolescents with the “Parents secure – Peers insecure” profile.

Interestingly, some studies also support the *peer compensatory hypothesis*, suggesting that secure attachment to peers when parental attachment is insecure can potentially protect against some adjustment problems. First, the person-oriented study by He et al. (2018) showed that adolescents with the “Parents insecure – Peers secure” profile had higher resiliency and optimism than those with the “Parents secure – Peers insecure” profile and better overall psychosocial adjustment than adolescents with the “All insecure” profile. Second, a variable-oriented study confirmed that adolescents with secure peer attachment showed high levels of prosocial behavior and empathy, even if they had insecure attachment to parents (Laible et al., 2000). Another study analyzed the reorganization of attachment hierarchy from parents to peers among Israeli male adolescents entering the army and found that those preferring peers as their attachment figures were more psychosocially adjusted, indicated by a sense of commitment, mastery, and concurrence with military ideals (Mayseless, 2004). Third, a study reported that secure peer attachment decreased the likelihood of being bullied at school in general, and especially among boys with insecure parental attachment (Murphy et al., 2017). It seems that peer attachments may have specific functions, different from attachment to parents, in enhancing adolescents’ good social adjustment.

The few studies that have examined adolescent attachment beyond the categorization of parents and peers suggest that specific attachments towards the mother and father as well as towards a friend and romantic partner may play unique roles. Insecure attachment to the mother rather than to the father is commonly associated with mental health problems of depression and anxiety (Margolese et al., 2005; Keizer et al., 2019), whereas insecure attachment to the father may play a greater role in increasing adolescent risk-taking behavior such as harmful sexual relationships (Guilamo-Ramos et al., 2012). Furthermore, not all peer relationships seem equally important. Wilkinson (2010) showed that it was especially insecure attachment to the best friend that predicted high levels of depressive symptoms, negative attitudes towards school, and low self-competence, even after controlling for attachment to parents and other peers. Romantic attachments often become especially salient in late adolescence (Markiewicz et al., 2006) and insecure romantic attachments are in general associated with mental health problems (Mikulincer and Shaver, 2012). However, little is known about their relative contributions in attachment hierarchies regarding adolescent psychosocial adjustment, as romantic attachments are often grouped together with attachment to friends to represent general peer attachment. We could locate only one previous study (Margolese et al., 2005) analyzing the specific roles of adolescent attachment to parents, best friend, and romantic partner in terms of adolescent psychosocial adjustment. The study found insecure attachment to the partner to be uniquely predictive of depression.

### Research Questions

The first aim of this person-oriented study is to describe the profiles that capture the versatility of adolescent attachment relationship hierarchies. We use latent profile analysis to identify profiles based on attachment avoidance and anxiety in adolescents’ relationships with mother, father, best friend, and romantic partner. In line with two previous person-oriented studies (Wang and Wang, 2012; He et al., 2018), we expect to find at least four attachment profiles: “All secure” (low levels of anxiety and/or avoidance towards all attachment figures), “All insecure” (high levels of anxiety and/or avoidance towards all attachment figures), “Parents secure – Peers insecure,” and “Parents insecure – Peers secure.” Due to lack of previous research, we do not pose specific hypotheses regarding the relationships or the degree of avoidance and anxiety in the profiles.

Our second aim is to investigate how the identified profiles are associated with adolescent psychosocial adjustment, involving mental health (internalizing symptoms, inattention/hyperactivity symptoms, and anger control problems) and risk-taking behavior (substance use and sexual risk-taking). According to the *security-resilience* hypothesis on the beneficial role of multiple secure attachments, we expect adolescents in the “All secure” profile to show higher psychosocial adjustment (less mental health problems and risk-taking behavior) than adolescents in the other profiles. According to our *insecurity-vulnerability* hypothesis, we expect adolescents in the “All insecure” profile to show lower psychosocial adjustment (more mental health problems and risk-taking behavior) than the other profiles. Related to attachment figure-specific insecure profiles (i.e., involving some insecure attachment relationships), we propose two complementary hypotheses. According to the *parental primacy hypothesis*, we expect adolescents in the “Parents insecure – Peers secure” profile to show higher levels of mental health problems and risk-taking behavior than adolescents in the “Parents secure – Peers insecure” profile. According to the *peer compensation hypothesis*, emphasizing the protective role of secure peer relationships, we hypothesize that adolescents with the “Parents insecure – Peers secure” profile show lower levels of mental health problems and risk-taking behavior than those with the “All insecure” profile.

## Materials and Methods

### Participants and Procedure

The participants comprised 449 Finnish 17–19 year-old adolescents (*M*_*age*_ = 17.73, *SD* = 0.45), whose families had participated in the (Miracles of Development) longitudinal study from pregnancy through the child’s infancy and middle childhood to late adolescence. At the time of the child’s adolescence, 91.6% of mothers and 88.2% of fathers had permanent jobs or were entrepreneurs. The parents were relatively highly educated: 41.5% of mothers and 41.5% of fathers had a master’s degree and 31.2% of mothers and 24.3% of fathers had a polytechnic level degree (equivalent to a bachelor’s degree). The family income (reported by mothers) was relatively high, as 33.4% of families had a monthly income of 5000-7500e, 21.7% an income of 7500-10000e, and 18.1% an income of 3000-5000e.

The adolescents were approached by mailed letters (separately from their parents) informing them about the follow-up study, and they were asked to sign and return the informed consent in a prepaid envelope if interested in participating. Thereafter, the participating adolescents answered electronic questionnaires. The project complies with the Code of Ethics of the World Medical Association (1964 Declaration of Helsinki), and the Ethics Committee of Helsinki University Hospital approved the data collection protocol in late adolescence.

Attrition analysis utilizing previously collected family data (i.e., child’s gender, ART status, parents’ age, parents’ education level, and number of older siblings) showed some significant trends in missingness (Little’s MCAR (979) = 1077.75, *p* = 0.015). The participating adolescents were more often girls than boys (*Z* = −5.43, *p* < 0.001), and were from families with older fathers (*Z* = −2.02, *p* = 0.043), and higher maternal (*Z* = −2.76, *p* = 0.006), and paternal (*Z* = −4.11, *p* < 0.001) education level. No differences emerged between participating and non-participating adolescents in ART status, mother’s age, or number of older siblings.

### Measures

**Adolescents’ attachment relationships** with the mother, father, best friend, and romantic partner were assessed by the Experiences in Close Relationships – Relationship Structures (ECR-RS; Fraley et al., 2011). The measure was first translated to Finnish and then back-translated to English by a bilingual translator. The 9-item ECR-RS is a self-report questionnaire designed to assess attachment-related *Anxiety* (3 items; e.g., “I’m afraid that this person will abandon me”) and *Avoidance* (6 items; e.g., “I prefer not to show this person how I feel deep down”) in a relationship with the mother, father, best friend, and romantic partner (36 items in total). In case the adolescent did not have a current romantic partner, they were asked to report on a former partner, or in case they had never dated, on an imagined partner, according to standard instructions of the measure. Adolescents responded how well each description fit each relationship on a 7-point Likert-type scale (from 1 = *Strongly disagree* to 7 = *Strongly agree*). Separate scores were computed for the four relationships regarding avoidant attachment (mother α = 0.90, father α = 0.90, friend α = 0.88, and partner α = 0.87) and anxious attachment (mother α = 0.88, father α = 0.89, friend α = 0.91, and partner α = 0.93) dimensions. High scores on avoidant and anxious dimensions reflect high insecure attachment, and low scores on both dimensions indicate secure attachment. The validity of ECR-RS among adolescents has been demonstrated by showing meaningful associations with the more generalized attachment styles (Donbaek and Elklit, 2014).

**Mental Health Problems** were measured by the Self-Report of Personality — Adolescent (SRP—A) of the Behavior Assessment System for Children, Third Edition (BASC-3; Reynolds and Kamphaus, 2015). The measure was first translated to Finnish and then back-translated to English by a bilingual translator. In this study, adolescents’ *internalizing symptoms*, *inattention/hyperactivity symptoms*, and *anger control problems* were chosen to indicate mental health. Internalizing symptoms involve *Depression* (12 items; e.g., “I don’t seem to do anything right”), *Anxiety* (13 items; e.g., “I worry but I don’t know why”), and *Somatization* (7 items; e.g., “I have trouble breathing”) scales. Inattention/hyperactivity symptoms involve *attention problems* (8 items; e.g., “I am easily distracted”) and *hyperactivity* (8 items; e.g., “I have trouble sitting still”) scales. The Anger control problems scale describes a tendency to become irritated and inability to regulate one’s affect (10 items; e.g., “When I get angry, I want to hurt someone”). The adolescents used dichotomous (1 = *True* or 0 = *False*) and 4-point Likert-type scales (from 0 = *Never* to 3 = *Almost always*) to answer the questions, depending on the item. Sum scores (with higher scores indicating more severe symptoms) were computed for internalizing symptoms (α = 0.95), inattention/hyperactivity symptoms (α = 0.89), and anger control problems (α = 0.80).

**Substance use** was assessed by self-reported use of alcohol, nicotine products, and illegal drugs. Alcohol use was measured with the Consumption scale of the Alcohol Use Disorders Identification Test (AUDIT-C; Saunders et al., 1993; Bush et al., 1998). The official pre-existing Finnish version of the questionnaire was used. AUDIT-C assesses the frequency of alcohol use, the typical dose consumed on one occasion, and the tendency to drink over six doses on one occasion with a Likert scale (0 to 4). Following Kelly et al. (2009), a cut-off score of ≥5 for girls and ≥6 for boys was applied to indicate alcohol problem use (0 = not present, 1 = present) among young adults. The use of nicotine products and illegal drugs was assessed with items obtained from the Finnish School Health Promotion study (Halme et al., 2018). Three items assessed frequency of smoking regular and electronic cigarettes, and use of snuff tobacco, with a Likert scale (0 = *Not at all* to 4 = *Daily*). A cut-off score of >3 was applied such that daily use of some nicotine product indicated frequent use (0 = *Not present*, 1 = *Present*). The questions concerning illegal drug use assessed the times (0 = never, 3 = more than five times) one had consumed cannabis products, ecstasy/amphetamine, buprenorphine, other hard drugs (e.g., heroine/LSD/gamma) and alcohol to become intoxicated, huffed something in order to become intoxicated, or used unknown substances. A cut-off score of >0 was applied such that any use of the forementioned drugs indicated substance use (0 = *Not present*, 1 = *Present*). Finally, a count variable based on presence of alcohol problem use (0/1), daily use of nicotine products (0/1), and any use of illegal drugs (0/1) was computed to be used in the main analyses (range 0 to 3).

**Sexual risk-taking** was measured with the risky sexual behaviors subscale of the Cognitive Appraisal of Risky Events (CARE; Fromme et al., 1997). The measure was first translated to Finnish and then back-translated to English by a bilingual translator. The full CARE assesses adolescents’ outcome expectancies about the risks and benefits associated with involvement in risky activities in various domains (e.g., risky sports and illegal behaviors). The subscale utilized included six items that cover involvement in risky sexual activities (“Leaving a social event with someone I have just met”; “Sex without protection against pregnancy”; “Sex without protection against sexually transmitted diseases”; “Involvement in sexual activities without my consent”; “Sex with multiple partners”; “Sex with someone I have just met or don’t know well”). Participants indicated their involvement in these activities during the past 6 months on a 7-point Likert scale (0 = *Never*, 1 = Once, 2 = 2−4 times, 3 = 5−9 times, 4 = 10−20 times, 5 = 21−30 times, 6 = Over 31 times). Initially, we computed a variable by averaging responses to all of the items (M = 1.56, SD = 3.00), however, its distribution was highly skewed (skewness = 2.65, kurtosis = 8.78; α = 0.61). Thus, we dichotomized the responses (0 = *Never* and ≥ 1 = *Once or more*) and formed a count variable that more unanimously indicated the number of risky activities (range 0 to 6) in which one had engaged (α = 0.73).

**Background variables** were used as a covariates in the main analyses to ensure that the results were not biased by some common third factors. The variables were Adolescent gender (1 = *Girl*, 2 = *Boy*); Adolescent age (in years); Adolescent education (1 = *Lower education*, 2 = *High school*); Romantic relationship status (1 = *Single*, 2 = *Currently in a relationship*); Parents’ marital status (1 = *Together*, 2 = *Divorced*); and ART status (1 = *ART*, 2 = *NC*).

### Data Analysis Plan

To answer our first research question, we conducted a latent profile analysis (LPA) in Mplus 7.4 Muthén and Muthén (1998–2015) to identify distinct attachment profiles. LPA is a form of mixture modeling in which latent classes are derived based on means of the observed indicator variables. The indicator variables were the adolescent’s attachment anxiety and avoidance towards the mother, father, best friend, and romantic partner (8 variables). We used a censored-inflated model to take into account the highly skewed distributions of the indicator variables (skewness ranged for anxiety variables from 1.26 to 2.49, and for avoidance variables from 0.34 to 1.46). We decided the number of latent classes based on multiple criteria, e.g., Akaike’s Information Criterion (AIC), Bayesian Information Criterion (BIC), and the adjusted BIC (aBIC). In addition, we used the Bootstrapped Log Likelihood Ratio Test (BLRT; McLachlan and Peel, 2000) and the Vuong-Lo-Mendell-Rubin Test (VLMR; Lo et al., 2001) to test for the optimal number of classes. Finally, we considered both theoretical (i.e., interpretability and meaningfulness) and pragmatic (e.g., number and size of classes) aspects when deciding on the final number of latent classes (Nylund-Gibson and Choi, 2018). We used entropy statistic to describe clarity of the selected solution.

Following the recommendations of Nylund-Gibson et al. (2019), we used a manual three-step BCH method (Bakk et al., 2013) to include both covariates and distal outcomes in the selected latent model. The benefit of the BCH method is that it considers the classification error for each individual separately and is robust against changes in the classification that could result from the inclusion of auxiliary variables. In practice, we modeled the predictive effects of background variables (adolescent’s gender, age, education level, romantic relationship status, parents’ marital status, and ART status) on the latent classes and included these as covariates when testing the effects of the latent classes on adolescent psychosocial adjustment (internalizing, inattention/hyperactivity, and anger control symptoms as well as substance use and sexual risk-taking). Poisson regression was used for the count type sexual risk-taking (with zero-inflated probability distribution) and the substance use variables. Two-stage sharpened method (Benjamini et al., 2006) was applied to adjust the False Discovery Rate (FDR) for pairwise tests regarding the effects of latent classes on adolescent psychosocial adjustment (40 *p*-Values).

## Results

### Descriptive Statistics

More than half of the participating adolescents (*n* = 449) were girls (58.9%), and the rest were boys (39.4%) or transgenders (1.6%). As the group of transgenders was too small to be analyzed statistically, they were excluded from the analysis. Most participants (74.4%) had upper secondary education (or were students at this level), whereas the rest had a lower level of education. One-third (30.6%) were currently in a romantic relationship, one-third (29.7%) came from families in which the parents were divorced or separated, and one-half (49.1%) were conceived with ART. Regarding substance use, most (60%) of the participants had zero risk, 21% had one risk, 14% had two risks, and 5% had all three risks. Regarding sexual risk-taking, most (66%) of the participants had not engaged in risky activities, 15% had engaged in one risk, 9% in two risks, 6% in three risks, and the remaining 5% in four or more risks.

Concerning correlations between the study variables (see Table 1), boys (as compared with girls) tended to be more avoidant in their relationship with their mother, best friend, and partner. Adolescents with higher education level were less anxious in their relationship with their mother and father and less avoidant in their relationship with their best friend, and partner. Adolescents in a current romantic relationship were less avoidant in their relationship with their mother, best friend, and partner. Parental divorce correlated with an adolescent’s higher avoidant and anxious attachment with their father. Overall, both attachment anxiety and avoidance correlated with higher mental health problems and to some extent with substance use and sexual risk-taking. However, it must be noted that most correlations were small (| *r*| = 0.10−0.30).

**TABLE 1:** Descriptive statistics and correlations of the study variables.

	M	SD	1.	2.	3.	4.	5.	6.	7.	8.	9.	10.	11.	12.	13.	14.	15.	16.	17.	18.	19.
(1) ART status	1.51	0.50	1.00																		
(2) Parental divorce	1.30	0.46	0.07	1.00																	
(3) Adolescent age (in years)	17.73	0.45	0.34***	0.10*	1.00																
(4) Adolescent gender	1.41	0.49	0.03	0.07	–0.01	1.00															
(5) Adolescent relationship status	1.30	0.46	–0.01	0.12*	–0.06	–0.05	1.00														
(6) Adolescent education	1.80	0.40	0.05	−0.21***	–0.04	−0.25***	0.01	1.00													
(7) Mother anxiety	1.41	0.91	–0.03	0.07	0.09	0.03	–0.03	−0.12*	1.00												
(8) Mother avoidance	2.73	1.39	–0.01	0.07	0.09	0.11*	−0.14**	–0.02	0.36***	1.00											
(9) Father anxiety	1.60	1.10	–0.02	0.15**	0.09	–0.02	0.01	−0.11*	0.62***	0.25***	1.00										
(10) Father avoidance	3.49	1.52	0.12*	0.18***	0.16***	–0.04	–0.03	–0.02	0.28***	0.58***	0.51***	1.00									
(11) Friend anxiety	2.17	1.45	–0.05	0.02	0.04	–0.02	−0.10*	–0.02	0.42***	0.34***	0.39***	0.34***	1.00								
(12) Friend avoidance	2.18	1.13	–0.06	0.06	0.05	0.32***	−0.12*	−0.19***	0.21***	0.31***	0.23***	0.27***	0.37***	1.00							
(13) Partner anxiety	2.10	1.51	0.05	0.07	0.05	0.03	–0.07	–0.08	0.29***	0.20***	0.26***	0.20***	0.29***	0.14**	1.00						
(14) Partner avoidance	1.87	1.00	0.08	0.05	0.03	0.14**	−0.19***	−0.16***	0.25***	0.24***	0.29***	0.25***	0.21***	0.37***	0.54***	1.00					
(15) Sexual risk-taking	0.73	1.22	0.07	0.13**	0.15*	–0.03	0.09	–0.08	0.06	0.05	0.10*	0.13**	–0.01	0.06	0.17***	0.16***	1.00				
(16) Anger control problems	0.57	0.40	–0.02	0.14**	0.10*	–0.09	0.02	–0.07	0.34***	0.27***	0.29***	0.29***	0.34***	0.10*	0.23***	0.14**	0.22***	1.00			
(17) Inattention/hyperactivity	1.25	0.93	–0.01	0.11*	0.04	−0.13**	0.03	−0.12*	0.18***	0.16***	0.15**	0.22***	0.19***	–0.07	0.17***	0.09	0.13**	0.54***	1.00		
(18) Internalizing symptoms	1.72	1.33	0.02	0.04	0.09	−0.36***	–0.05	0.04	0.31***	0.30***	0.24***	0.36***	0.44***	0.09	0.21***	0.14**	0.13**	0.66***	0.52***	1.00	
(19) Drug abuse and dependency	0.65	0.92	0.03	0.15**	0.12*	0.15**	0.02	−0.19***	0.08	0.22***	0.09	0.22***	0.05	0.06	0.27***	0.20***	0.46***	0.23***	0.16***	0.12*	1.00

Values represent 1 = ART and 2 = NC for ART status; 1 = Together and 2 = Divorced for Parental Divorce; 1 = Girl and 2 = Boy for Child’s Gender; 1 = Single and 2 = In a relationship for Adolescent Relationship status; 1 = Lower education and 2 = High school for Adolescent Education. *p < 0.05; **p < 0.01; and ***p < 0.001.

We tested how the background variables together were associated with an adolescent’s psychosocial adjustment using a regression analysis (for detailed results, see Supplementary Table 1). In summary, boys experienced less internalizing symptoms, less inattention/hyperactivity symptoms, and less anger control problems, but more substance use than girls. An adolescent’s older age was associated with more anger control problems, substance use, and sexual risk-taking. An adolescent’s low education level was associated with more inattention/hyperactivity symptoms, substance use, and sexual risk-taking. Finally, parents’ divorce was associated with an adolescent’s higher anger control problems and substance use. An adolescent’s current romantic relationship or ART status did not predict mental health or risk-taking behaviors. These effects were moderate (| *β’s*| = 0.24 to 0.37) in magnitude.

### Latent Attachment Profiles

To answer our first research question on adolescents’ latent attachment profiles, as shown in Table 2, the VLMR suggested that the optimal solution involves either two, four, or five latent classes. However, neither fit indices (AIC, aICC, BIC, aBIC) nor the BLRT preferred a solution within a range from one to seven latent classes. As we had expected at least four latent classes to emerge, we examined the theoretical interpretability of the four- and five-class solutions. All of the classes in the four-class solution were substantially replicated in the five-class solution, with one theoretically interesting class (i.e., third class: “Parents secure – Partner insecure”) emerging in the five-class solution. Thus, we chose to use the five-class solution. It provided reasonable group sizes (ranging from 43 to 175) and good entropy (0.84), indicating distinctiveness of the classes. Lack of a unanimous solution in LPA may indicate strong heterogeneity in the attachment profiles. Despite this, the classes allow condensing a large amount of information into a manageable number of classes.

**TABLE 2:** Summary of different class solutions from latent profile analysis (LPA).

Number of Classes	LL	AIC	aICC	BIC	aBIC	Entropy	VLMR *p*-Value	BLRT *p*-Value	Class size
1	−5332.94	10697.88	10699.15	10763.45	10712.67	NA	NA	NA	445
2	−5015.28	10080.56	10083.66	10183.01	10103.67	0.80	0.000	0.000	259/186
3	−4928.12	9924.23	9930.04	10063.57	9955.67	0.80	0.170	0.000	209/157/79
4	−4872.38	9830.77	9840.20	10006.98	9870.52	0.85	0.030	0.000	202/145/56/42
5	−4818.59	9741.17	9755.24	9954.27	9789.25	0.84	0.020	0.000	175/95/84/48/43
6	−4785.12	9692.25	9712.00	9942.23	9748.64	0.85	0.800	0.000	178/86/77/44/41/19
7	−4754.28	9648.55	9675.13	9935.42	9713.27	0.85	0.370	0.000	160/94/74/45/37/7

*LL, Log Likelihood; AIC, Akaike’s Information Criterion; aICC, Adjusted Akaike’s Information Criterion; BIC, Bayesian Information Criterion; aBIC, Adjusted Bayesian Information Criterion; VLMRT, Vuong-Lo-Mendell-Rubin Test; BLRT, Bootstrapped Log Likelihood Ratio Test.*

**FIGURE 1:**
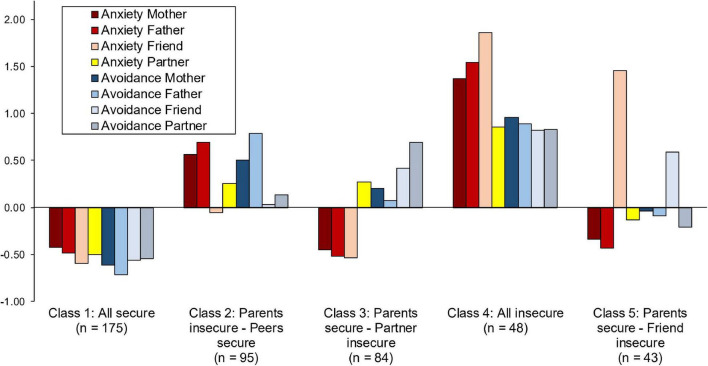
Standardized means of indicator variables for adolescent latent attachment profiles.

Table 3 presents indicator variable means and Figure 1 standardized variable means for each profile in the five-class solution. The first profile comprised 39% of the sample and involved low scores on both attachment anxiety and avoidance in all relationships. Thus, we labeled it as an “All secure” attachment profile. The second profile (21%) involved high attachment anxiety and avoidance scores in maternal and paternal relationships, but not in peer relationships (i.e., friend and romantic partner). Thus, we labeled it as a “Parents insecure – Peers secure” attachment profile. The third profile (19%) involved low attachment anxiety within mother, father, and friend relationships, and high avoidance especially in romantic partner relationships. Thus, we labeled it as a “Parents secure – Partner insecure” attachment profile. The fourth profile (11%) involved high scores in both attachment anxiety and avoidance in all relationships. Thus, we labeled it as an “All insecure” attachment profile. Finally, the fifth profile (10%) involved low attachment anxiety within mother and father relationships, low avoidance in partner relationships, and especially high attachment anxiety and avoidance in friend relationships. Thus, we labeled it as a “Parents secure – Friend insecure” attachment profile.

**TABLE 3:** Means of indicator variables for adolescent latent attachment classes.

		Class 1 (*n* = 175):All secure		Class 2 (*n* = 95): Parents insecure – Peers secure		Class 3 (*n* = 84): Parents secure – Partner insecure		Class 4 (*n* = 48):All insecure		Class 5 (*n* = 43):Parents secure – Friend insecure		Between-group effects (Wald’s test)
							
		*M*	*SD*	*M*	*SD*	*M*	*SD*	*M*	*SD*	*M*	*SD*	
Anxiety	Mother	1.02^a^	0.01	1.86^b^	0.89	1.00^d^	0.94	2.75^c^	2.27	1.15^a^	0.25	123.67
	Father	1.08^a^	0.28	2.33^b^	1.18	1.04^a^	0.17	3.30^c^	1.39	1.14^a^	0.44	210.04
	Friend	1.28^a^	0.48	2.09^b^	0.75	1.37^a^	0.50	4.86^c^	0.96	4.28^c^	0.86	603.90
	Partner	1.36^a^	0.86	2.41^bc^	1.41	2.53^bc^	1.71	3.39^d^	1.67	2.02^ac^	1.6	33.89
Avoidance	Mother	1.82^a^	0.67	3.42^c^	1.86	3.01^bc^	1.38	4.06^d^	1.86	2.68^b^	1.68	169.52
	Father	2.37^a^	0.98	4.67^c^	1.25	3.60^b^	1.07	4.84^c^	1.27	3.36^b^	1.41	189.08
	Friend	1.50^a^	0.64	2.20^b^	1.03	2.66^bc^	1.19	3.11^c^	1.20	2.83^c^	1.07	131.63
	Partner	1.29^a^	0.48	1.96^b^	0.83	2.54^c^	1.10	2.71^c^	1.29	1.66^b^	0.80	90.21

*Different superscripts (a−d) in each row indicate statistically significant, p < 0.05, differences between the latent classes. All Wald’s tests had 4 degrees of freedom and indicated significant, p < 0.05, differences between the groups.*

### Background Variables and Latent Attachment Profiles

To examine how the background variables predicted class membership in the latent attachment profiles, we used logistic regression from the BCH analysis. The results showed that boys were more likely than girls to belong to the “Parents secure – Partner insecure” profile compared with the other profiles: “All secure,” β = −0.65, *B* = −1.32, *SE* = 0.40, *p* = 0.001, “Parents insecure – Peers secure,” β = −0.55, *B* = −1.13, *SE* = 0.42, *p* = 0.007, “All insecure,” β = −0.47, *B* = −0.95, *SE* = 0.48, *p* = 0.045, and “Parents secure – Friend insecure,” β = −0.51, *B* = −1.05, *SE* = 0.49, *p* = 0.033. Furthermore, adolescents in a current relationship were more likely to belong to the “All secure” profile than the “Parents secure – Partner insecure” profile, β = 0.49, *B* = 1.07, *SE* = 0.49, *p* = 0.031. Adolescent’s age, education level, parental marital status, or ART did not predict the latent profile membership.

### Latent Attachment Profiles and Psychosocial Adjustment

**FIGURE 2:**
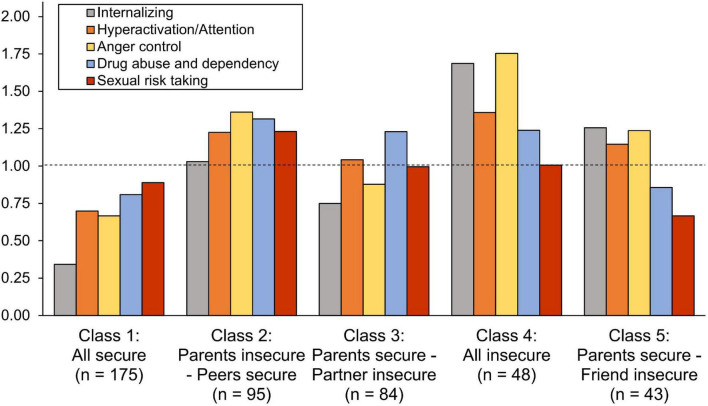
Mean estimates of mental health problems and risks for adolescent latent attachment profiles. The bars represent standardized (M = 1.0, SD = 1.0) scores with mean level indicated by dotted line.

To answer our second research questions regarding the associations of latent attachment profiles with adolescent psychosocial adjustment (mental health and risk-taking), we first conducted omnibus tests in the BCH analysis (see Between-group effects in Table 4). The results showed that adolescents from different latent attachment profiles differed in internalizing symptoms, inattention/hyperactivity symptoms, anger control problems, and substance use. Against our hypothesis, there were no differences in sexual risk-taking. The mean differences between the latent attachment profiles are shown in Figure 2 and Table 4. All background variables were used as covariates in the analysis.

**TABLE 4:** Estimated means for mental health problems and risk-taking behaviors among adolescent latent attachment classes.

	Class 1 (*n* = 175): All Secure		Class 2 (*n* = 95): Parents insecure – Peers secure		Class 3 (*n* = 84): Parents secure – Partner insecure		Class 4 (*n* = 48): All insecure		Class 5 (*n* = 43): Parents secure – Friend insecure		Between-group effects (χ2)	
						
	*M*	*SE*	*M*	*SE*	*M*	*SE*	*M*	*SE*	*M*	*SE*	χ2	*p*
Internalizing symptoms	0.88	0.20^a^	1.79	0.22^bd^	1.42	0.23^b^	2.67	0.28^c^	2.09	0.29^cd^	90.15	<0.001
Inattention/hyperactivity symptoms	1.00	0.16^a^	1.48	0.16^b^	1.31	0.19^b^	1.61	0.20^b^	1.41	0.23^ab^	22.17	<0.001
Anger control problems	0.45	0.06^a^	0.72	0.07^b^	0.53	0.08^ad^	0.88	0.10^b^	0.67	0.09^bd^	47.47	<0.001
Substance use	0.50	0.16^a^	0.95	0.19^b^	0.73	0.06^bc^	0.88	0.21^bc^	0.54	0.16^ac^	13.52	0.009
Sexual risk-taking	0.44	0.07	0.95	0.19	1.39	0.14	0.76	0.27	0.35	0.26	3.06	0.548

*Different superscripts (a−d) in each row indicate statistically significant, p < 0.05, differences between the latent classes with FDR adjustments. All χ^2^ tests had 4 degrees of freedom.*

In line with our *security-resilience* hypothesis, *post hoc* tests (see Table 4) showed that adolescents in the “All secure” profile, as compared with all other profiles, experienced lower internalizing symptoms, inattention/hyperactivity symptoms, anger control problems, and substance use; however, two exceptions emerged in the pairwise tests. Against our hypothesis, the “All secure” profile did not differ in anger control problems from the “Parents secure – Partner insecure” profile, or in substance use from the “Parents secure – Friend insecure” profile.

Consistent with our *insecurity-vulnerability* hypothesis, adolescents in the “All insecure” profile reported the highest mean levels of internalizing symptoms, inattention/hyperactivation symptoms, and anger control problems. However, the differences were statistically significant only with some of the other profiles involving insecurity: the “All insecure” profile had higher internalizing symptoms than the “Parents insecure – Peers secure” and “Parents secure – Partner insecure” profiles. Furthermore, the “All insecure” profile had more anger control problems than the “Parents secure – Partner insecure” profile. Against our hypothesis, there were no differences in the amount of inattention/hyperactivity symptoms and substance use between the “All insecure” profile and the other profiles involving insecurity.

In accordance with the *parental primacy hypothesis*, adolescents in the “Parents insecure – Peers secure” profile had higher anger control problems than those in the “Parents secure – Partner insecure” profile, and adolescents in the “Parents insecure – Peers secure” had higher substance use than those in the “Parents secure – Friend insecure” profile. Against our hypothesis, however, there were no differences in internalizing symptoms, inattention/hyperactivity symptoms, or substance abuse between these partially insecure attachment profile groups.

In line with our *peer compensation hypothesis*, adolescents in the “Parents insecure – Peers secure” profile had lower internalizing symptoms than adolescents in the “All insecure” profile. However, there were no differences in the other indicators of adolescent psychosocial adjustment between the attachment profile groups.

Finally, as an additional analysis we focused on comparisons between the two new latent profiles found in our study, with secure attachment towards both parents but insecurity towards either the best friend or romantic partner. The single difference was that adolescents in the “Parents secure – Friend insecure” profile had higher internalizing symptoms than those in the “Parents secure – Partner insecure” profile.

## Discussion

We analyzed the emergence of relationship-specific attachment profiles in late adolescence and their associations with adolescent psychosocial adjustment, comprising mental health problems and risk-taking behavior. Person-oriented LPA identified five distinct attachment profiles, reflecting the multitude of attachment figures of father, mother, and peers (here best friend and romantic partner) and attachment security and insecurity towards them. The identified profiles partially concur with earlier person-oriented research (He et al., 2018) and variable-oriented group analyses (Laible et al., 2000; Laghi et al., 2009) in revealing “All secure,” “All insecure,” and “Parents insecure – Peers secure” profiles. However, instead of a unified “Parents secure – Peers insecure” profile, we found two distinct profiles where attachment to only one peer was clearly insecure: “Parents secure – Friend insecure” and “Parents secure – Partner insecure.” The attachment profiles were associated with adolescents’ psychosocial adjustment, providing partial, outcome-specific support for our hypotheses. Consistent with the *security-resilience hypothesis* and the *insecurity vulnerability hypothesis*, adolescents in the “All secure” profile showed the highest psychosocial adjustment, whereas adolescents in the “All insecure” profile tended to show low adjustment. Consistent with the *parental primacy hypothesis*, adolescents in the “Parents insecure – Peers secure” profile showed more anger control and substance use problems than adolescents in the other partially insecure profiles, where parental attachment was secure. Results also partially supported the *peer compensation hypothesis* regarding internalizing symptoms, as adolescents in the “Parents insecure – Peers secure” profile showed less symptoms than adolescents in the “All insecure” profile. Our findings help both understand the organization of attachment hierarchies in adolescence and refine the role of specific attachment relationships in psychosocial adjustment.

### Versatility of Adolescents’ Attachment Profiles

“All secure” attachment was the most prevalent of our five identified profiles, comprising 39% of all profiles and involving equally low attachment anxiety and avoidance across all significant relationships. By contrast, the share of “All insecure” attachment was only 11%, and the profile was characterized by heterogeneous levels of insecurity, manifested in very high anxious attachment to parents and friends and relatively high avoidant attachment to all significant others. The profile distribution concurs with meta-analyses showing a higher share of secure (58%) than insecure (23% dismissing/avoidant and 19% preoccupied/anxious) attachment styles in Western countries (Bakermans-Kranenburg and van IJzendoorn, 2009). Yet, our person-oriented findings emphasize the complexity and relationship-specificity of attachment hierarchies, making it less meaningful to describe attachments simply along the lines of security versus insecurity. The high level of anxious attachment in the “All insecure” profile is noteworthy, as meta-analyses reveal attachment anxiety to form more severe risks for mental health and development than attachment avoidance (Korver-Nieberg et al., 2014; Dagan et al., 2020), apparently due to its low fit with societal demands emphasizing high self-management.

It is noteworthy that attachment security or insecurity to mothers and fathers did not differ in any of the identified profiles, which may reflect developmentally apt shaping of attachment hierarchies, characterized by transfer towards increasingly integrated representations of different familial attachment figures in late adolescence (Bowlby, 1980).

The emergence of “All secure” and “All insecure” profiles is in accord with the generalization or symmetry principle in attachment development; that is, the internal working models of self-worth, benevolence of others, and environmental safety, learned within the family, are transferred towards other significant relationships, here to best friends and romantic partners (Bowlby, 1980; Bretherton and Munholland, 2016). The asymmetric profiles showing insecurity towards the best friend (10%) or romantic partner (19%), while having a secure parental relationship may in turn reflect a staggered or slow transition and hierarchical dynamics in late adolescence.

Our results confirm that in late adolescence it is informative to separately assess attachments towards best friends and romantic partners, as adolescents use multiple attachment figures to satisfy their specific needs. Friends contribute to social skills, identity calibration, and need for disclosure, and romantic partners are pivotal to proximity seeking and emotional and sexual intimacy, yet, both attachments serve as training ground for mature adulthood attachments (Bowlby, 1980; Markiewicz et al., 2006; Kansky and Allen, 2018; Fraley, 2019). The transition of parents as a secure base and safe haven first to friends and then to romantic partners is crucial for optimal development (Weiss, 1991; Markiewicz et al., 2006). Our study did not examine the reasons for insecure attachment towards friends or romantic partners. However, for example bullying or discrimination experiences can result in insecure peer attachments (Murphy et al., 2017), and inadequate conflict resolution and emotion regulation strategies can lead to insecure partner attachment (Jorgensen-Wells et al., 2021).

The hierarchical attachment profiles identified among Finnish adolescents were to some extent similar to those found in two earlier person-oriented studies among Chinese adolescents, although the distributions differed. In our study, 39% of adolescents showed the “All secure” attachment profile, a figure situated between those of two earlier studies (66% in Wang and Wang, 2012 and 27% in He et al., 2018). In our study, fewer adolescents (10%) showed the “All insecure” profile than in He et al. (25%). Wang and Wang did not identify a pure “All insecure” profile, but their “Average” profile (18%) seemed to capture some generalized insecurity.

Importantly, these differences in the attachment profile distribution between European and Asian adolescents relate to the question about the universal versus culture-bound nature of attachment (Keller, 2013; Mesman et al., 2016). The universal view follows an evolutionary explanation that in the face of distress and danger infants (and adults) seek proximity and rely on specific figures for safety (Bowlby, 1969). A review on parent-infant attachment in different cultures and geographic continents showed a similar share of securely attached children (a majority), but found high cultural variations in the shares of insecure-avoidant and insecure-anxious attachments (Mesman et al., 2016). Multiethnic observations in turn support the culture-bound nature of attachment by suggesting that cultures decisively differ in the extent of parenting values and socialization goals aimed at enhancing children’s autonomy, independence, and sense of self, or, alternatively, at cherishing relatedness, dependence, and sense of social harmony (Kagitcibasi, 2005; Keller, 2007, 2013). Also, research shows cross-cultural variation among adults in the emergence of secure, anxious, and avoidant attachments (Schmitt et al., 2004). It would be informative to further examine the extent to which adolescents’ multiple, hierarchically organized attachment profiles are universal or bound to culture.

Adolescent gender and current romantic relationships were associated with the profiles. Boys more often than girls belonged to the “Parents secure – Partner insecure” profile. This may indicate boys’ later onset of dating or transferring proximity seeking from parents to partners (Markiewicz et al., 2006) or reflect the evolution psychology explanation of avoidance being a part of a low-investment, low-commitment strategy considered more advantageous to males (Del Giudice, 2011). Currently dating adolescents more likely belonged to the “All secure” than the “Parents secure – Partner insecure” profile. This may illustrate normative and successful transfer of proximity and sharing towards partnership, while still preserving security in parents and friends (Markiewicz et al., 2006).

### Attachment Profiles and Adolescent Psychosocial Adjustment

In line with the *security-resilience hypothesis*, adolescents in the “All secure” attachment profile showed lower levels of mental health problems and substance use than those in any other profile. A comprehensive attachment security thus provides added benefits for adolescents. The theory of broadening and building positive emotions (Fredrickson, 2013; Mikulincer and Shaver, 2020) may explain developmental dynamics underlying the “All secure” profile. Experience of self as worthy, others as emotionally available in distress, and shared creation of safety broadens cognitions, emotions, and social resources, maintaining sense of security and trust in being beloved and capable of giving love and widening effective functioning in stressful encounters (Fredrickson, 2013). It is noteworthy that adolescents in the “All secure” profile showed the lowest levels of internalizing symptoms relative to all other profiles. Adolescence is a risk period for depression and anxiety (Costello et al., 2011), and the role of complete access to secure relationships in potentially preventing these mental health problems is critical.

In accordance with the *insecurity vulnerability hypothesis*, adolescents in the “All insecure” attachment profile showed higher levels of mental health problems and substance use than those in the “All secure” profile. Internalization symptoms and anger control problems were exceptionally high in the “All insecure” profile (Figure 2), and the profile also differed from partially insecure profiles in these dimensions by showing more internalization problems than “Parents secure – Partner insecure” and “Parents insecure – Peers secure” profiles and more anger control problems than the “Parents secure – Partner insecure” profile. These differences may reflect the psychological pain that adolescents who have no one to turn to direct onto themselves as depression or outwards as aggressive behavior. However, there were no differences in inattention/hyperactivity symptoms or risk-taking behavior compared with partially insecure profiles, suggesting that insecurity experienced in any significant relationship can severely interfere with adolescents’ optimal psychosocial adjustment.

We posed two complementary hypotheses concerning adolescent psychosocial adjustment in case of partially secure attachment hierarchies: the *parental primacy hypothesis*, assuming parental insecurity to form the main risk for adolescent psychosocial adjustment irrespective of peer attachment quality, and the *peer compensatory hypothesis*, suggesting that secure attachment to peers (best friend and/or romantic partner) may protect adolescents’ psychosocial adjustment despite parental insecurity. Our results indicated partial support for both, yet more strongly for the *parental primacy hypothesis*. Accordingly, adolescents in the “Parents insecure – Peers secure” profile showed more substance use than those in the “Parents secure – Friend insecure” profile and more anger control problems than those in the “Parents secure – Partner insecure” profile. Adolescents who lack security from parents seek it from their peers and may therefore need to prematurely organize their attachment hierarchies to include romantic partners or to have complete reliance on friends (Friedlmeier and Granqvist, 2006). Subsequently, they are driven to depend on attachment figures that may themselves still be immature, unstable, and unable to provide safety. Seeking security may also lead to affiliation with more deviant peers who use substances or are aggressive (McKay, 2015; Abbott et al., 2019).

The *peer compensatory hypothesis* was confirmed only concerning internalizing symptoms, as adolescents with the “Parents insecure – Peers secure” attachment profile showed less symptoms than those in the “All insecure” profile. Adolescents may find it easier to share emotional problems with friends than with parents, and therefore, internalizing symptoms can be more susceptible to the effective buffering of peer relations. Good peer relations can protect from depression and anxiety and generally promote good adolescent mental health (La Greca and Harrison, 2005). This also concurs with previous findings on the unique importance of insecure attachment to the best friend (Wilkinson, 2010) or romantic partner (Margolese et al., 2005) in predicting depression.

Our results emphasize the importance of separately investigating attachment to the best friend and romantic partner, as they serve specific functions in late adolescence. Interestingly, adolescents with insecure attachment to their best friend showed more internalizing symptoms than those with romantic partner insecurity. Possibly, attachment to a best friend at this age represents a more long-term, unique relationship, where problems are especially harmful for one’s well-being. Our study was the first to identify distinct profiles with romantic partner or best friend insecurity within an otherwise secure attachment hierarchy and to detect their specific impacts on internalizing symptoms.

Contrary to earlier findings on adolescent attachment insecurity increasing sexual risk-taking (Guilamo-Ramos et al., 2012; Sentino et al., 2018), attachment profiles were not associated with adolescent sexual risk-taking in our study. It is possible that factors other than attachment relationships may explain the development of sexual risk-taking. A community study found that familial attachment, social support, parental involvement, and self-esteem all affected sexual risk-taking only indirectly through substance use (Hamme et al., 2010). Other authors have found that insecure parental attachment was associated with sexual risk-taking only among girls and those with previous sexual experiences (Feeney et al., 2000; McElwain et al., 2015).

### Strengths and Limitations of the Study

Strengths of our study include a relatively large sample size and use of the person-oriented approach that can summarize complex attachment relationship patterns, which have rarely been investigated before. Limitations are related to, first, the statistical indicators that were ambiguous in suggesting the optimal number of latent attachment profiles. While such a situation is not uncommon in LPA, it leaves open the possibility that the latent model does not suit the data sufficiently well (Nylund-Gibson and Choi, 2018). In other words, adolescent attachment hierarchies may be so complex that they require a very high number of latent profiles (and larger sample sizes) to be reliably depicted. However, the latent profiles identified in our study were theoretically interesting, converged to some extent with previous person-oriented studies (Wang and Wang, 2012; He et al., 2018), and importantly, were associated meaningfully with an adolescent’s psychosocial adjustment. Second, our sample among late adolescents had biased drop-out from earlier assessments concerning especially a lower participation of boys, children with older fathers, and families with low parental education. Overall, the participating adolescents represented a relatively advantaged normative sample, where severe substance use, clinical mental health disorders, or strong sexual risk-taking were not common. Subsequently, the results can be generalized only to relatively normative low-risk samples. Third, our choice of self-report questionnaires warrants some critique. In particular, the sexual risk-taking questionnaire assessed a wide range of behaviors (and events), some of which may have been less ideal when used with adolescents approaching adulthood. For example, “having sex without protection against sexual diseases” can be a norm among couples in a committed relationship. Moreover, the item “participating in sexual intercourse without my consent” may reflect victimization instead of risky behavior. A focus on more theoretically driven aspects of sexual behavior may be important to capture attachment-related phenomena.

## Conclusion

In sum, adolescents have multiple attachment relationships that can differ in the degree of security and insecurity towards parents, friends, and romantic partners. Clearly, secure attachment to parents provides an especially salient resource still in late adolescence, with benefits on adolescent mental health and risk-taking behavior. The benefits of secure attachment to friends and romantic partners appear to be less clear; yet secure attachment to peers in the context of insecure parental attachment seem to buffer from emotional problems. Our results encourage further research to look into different profiles of attachment relationships to understand their specific roles in adolescent mental health and development.

Understanding the unique ways that adolescents organize their multiple attachment relationships can help professionals to support and treat individual adolescents with psychosocial or psychiatric problems. In particular, it is important to understand the vital role of parental emotional support still in late adolescence and early adulthood, as, according to our results, closeness and support from friends and romantic partners cannot replace parental security. In situations where support from parents is not available, therapeutic alliance may be instrumental in providing parent-like emotional security for the adolescent.

## Data Availability Statement

The datasets analyzed for this study are available from the authors upon request. Participant privacy and ethical permissions related to this data do not allow public sharing of the data. Requests to access the datasets should be directed to R-LP, raija-leena.punamaki@tuni.fi.

## Ethics Statement

The studies involving human participants were reviewed and approved by the Ethic Committee of Helsinki University Hospital. Written informed consent for participation was not provided by the participants’ legal guardians/next of kin because: Finnish legislation or ethics committee does not require parental permission from 17 − 18 year-olds, they are allowed to give their own informed consent. Parents were informed about their adolescents’ participation and also had themselves participated in this 18-year longitudinal study during several different time points, including now when their children were 17−18 years-old. Each family member decided about their own participation and provided their own informed consent.

## Author Contributions

MF, MV, and R-LP wrote and planned the manuscript, and carried out the funding acquisition. LH wrote the manuscript and carried out the data collection. AT and PP wrote and planned the manuscript. JL wrote and planned the manuscript, and carried out the analyses and funding acquisition. All authors contributed to the article and approved the submitted version.

## Conflict of Interest

The authors declare that the research was conducted in the absence of any commercial or financial relationships that could be construed as a potential conflict of interest.

## Publisher’s Note

All claims expressed in this article are solely those of the authors and do not necessarily represent those of their affiliated organizations, or those of the publisher, the editors and the reviewers. Any product that may be evaluated in this article, or claim that may be made by its manufacturer, is not guaranteed or endorsed by the publisher.
