# Supplementary material for: Adolescent Attachment Profiles Are Associated With Mental Health and Risk-Taking Behavior
Source: Front Psychol. 2021 Dec 2;12:761864. doi: 10.3389/fpsyg.2021.761864 (PMC8674949; doi:10.3389/fpsyg.2021.761864)
Supplement: Supplementary file 1 [file Data_Sheet_1.docx]

Table S1. Effects of Background Variables on Adolescent Psychosocial Adjustment.

|  | Internalizing  symptoms | | | |  | | Inattention/hyperactivity  symptoms | | | |  | Anger control  problems | | | |  | Substance  use | | | |  | Sexual  risk-taking | | | |
| --- | --- | --- | --- | --- | --- | --- | --- | --- | --- | --- | --- | --- | --- | --- | --- | --- | --- | --- | --- | --- | --- | --- | --- | --- | --- |
|  | *β* | *B* | *SE* | *p* | |  | *β* | *B* | *SE* | *p* |  | *β* | *B* | *SE* | *p* |  | *β* | *B* | *SE* | *p* |  | *β* | *B* | *SE* | *p* |
| ART status | 0.00 | 0.01 | 0.13 | .952 | |  | -0.01 | -0.02 | 0.10 | .802 |  | -0.05 | -0.04 | 0.04 | .328 |  | -0.03 | -0.07 | 0.14 | .623 |  | -0.05 | -0.10 | 0.14 | .475 |
| Adolescent  relationship status | -0.06 | -0.18 | 0.13 | .155 | |  | 0.03 | 0.06 | 0.10 | .548 |  | 0.01 | 0.01 | 0.04 | .860 |  | 0.03 | 0.06 | 0.14 | .696 |  | -0.08 | -0.18 | 0.13 | .166 |
| Parental divorce | 0.07 | 0.21 | 0.14 | .128 | |  | 0.09 | 0.18 | 0.10 | .075 |  | 0.13 | 0.12 | 0.05 | .009 |  | 0.13 | 0.28 | 0.14 | .045 |  | 0.09 | 0.20 | 0.13 | .138 |
| Adolescent gender | -0.37 | -1.02 | 0.12 | .000 | |  | -0.17 | -0.31 | 0.09 | .000 |  | -0.14 | -0.11 | 0.04 | .007 |  | 0.18 | 0.37 | 0.14 | .009 |  | -0.12 | -0.25 | 0.14 | .074 |
| Adolescent age  (in years) | 0.08 | 0.25 | 0.15 | .087 | |  | 0.04 | 0.09 | 0.11 | .442 |  | 0.12 | 0.11 | 0.05 | .022 |  | 0.16 | 0.36 | 0.18 | .043 |  | 0.24 | 0.53 | 0.16 | .001 |
| Adolescent education | -0.02 | -0.06 | 0.16 | .708 | |  | -0.12 | -0.28 | 0.13 | .027 |  | -0.05 | -0.05 | 0.05 | .320 |  | -0.15 | -0.36 | 0.15 | .016 |  | -0.13 | -0.32 | 0.15 | .035 |

*Note*. Values are 1 = ART and 2 = NC for ART status; 1 = Together and 2 = Divorced for Parental Divorce; 1 = Girl and 2 = Boy for Child’s Gender; 1 = Single and 2 = In a relationship for Adolescent Relationship status; 1 = Lower education and 2 = High school for Adolescent Education.

Table S2. A summary of the studies on adolescent attachment hierarchies and their role in psychosocial adjustment reviewed in the article.

| *Author(s) (year)* | *Study sample and design* | *Attachment measure(s)* | *Attachment relationships measured* | *Main variables* | *Approach to analyses* | *Main finding(s)* |
| --- | --- | --- | --- | --- | --- | --- |
| Abbott et al. (2019). | 129 clinical 12–18-year- old adolescents in treatment for suicidal ideation; data from baseline of a longitudinal randomized clinical trial | The Important People Interview | Four most important people in one’s life and four additional peers | Attachment, deviant peer affiliation, substance dependence, suicidal ideation, and depression | Social network approach | Youth who affiliated with deviant peers were more likely to identify peers rather than adults as attachment figures, and to report higher suicidal ideation. |
| Allen et al. (1996) | A clinical group (n=66) and a comparison group (n=76) of 25-year-olds from a longitudinal study. The clinical group was psychiatrically hospitalized at the age of 14 years. | Adult Attachment Interview | One generalized attachment representation, mainly based on childhood attachment to mother and father | Attachment, psychological distress, self-worth, antisocial behavior | Variable-oriented study | Nearly all previously hospitalized adolescents showed insecure adult attachment at the age of 25 years. |
| Freeman & Almond (2010) | 1012 US university students (18-45 years, M_age_ 20.4) in a cross-sectional design | Attachment Network Questionnaire | Mother, father, best friend, and romantic partner | Attachment, social support, parental approval, sexual experiences | Mixed method study (qualitative + variable-oriented quantitative) | Younger students who were not romantically or sexually involved were more likely to report their father as an attachment figure. |
| Freeman & Brown (2001) | 99 US 16–18-yearold high school students in a cross-sectional design | -Adolescent Separation Anxiety Test  -Nomination of primary attachment figure  -Attachment Support Inventory | Mother, father, best friend, and romantic partner | Attachment style, primary attachment figure, attachment support | Variable-oriented study | Parents and peers were equally likely to be nominated as primary attachment figures, but adolescents with insecure attachment style more often nominated peers as their primary figures. |
| Friedlmeier & Grankvist (2006) | Swedish (n=171) and German (n=178) 14–18-year-old adolescents in a longitudinal study over a 12–15month interval | -Adult Attachment Scales (modified)  -Hazan and Shaver’s (1987) three attachment history paragraphs | Mother, father, and an unspecified peer (“a person I like very much”) | Attachment to peers and parents, attachment functions, current romantic relationship | Variable-oriented study | Older adolescents, those in romantic relationship, and those with insecure attachment to mother were more likely to transfer their attachment from parents to peers. |
| Guilamo-Ramos et al. (2012) | Papers from 1980 to 2011 were searched related to adolescent (11-18 years) attachment to father and adolescent sexual behavior | Varying measures of paternal parenting processes or attachment | Fathers; in some of the studies, also mothers were included | Paternal parenting processes and adolescent sexual behaviors | Structured literature review | Fathers influence the sexual behavior of their adolescent children. |
| He et al. (2018) | 941 Chinese students (M_age_ 15.89 years) in a cross-sectional design | The Inventory of Parent and Peer Attachment | Parent and peer attachment | Attachment, resiliency, hope, optimism, life satisfaction, psychological distress | Person-oriented study | Four profiles were found: All secure, All insecure, Secure parent and insecure peer, and Insecure parent and secure peer. The All insecure profile showed the lowest adjustment, and the All secure profile the highest adjustment. The Insecure parent and secure peer profile showed more resiliency but also more distress than the Secure parent and insecure peer profile. |
| Keizer et al. (2019) | 542 Dutch adolescents (M_age_ 13.6 years at baseline) from a longitudinal three-wave cohort study | The Inventory of Parent and Peer Attachment Revised | Parent and peer attachment | Attachment, self-esteem, perceived parental relationship quality, friendship and romantic relationship characteristics | Variable-based study | Changes in attachment to parents were associated with changes in self-esteem, but changes in peer attachment were not predictive of changes in self-esteem. |
| Laghi et al. (2009) | 2665 Italian high school students (M_age_ 17.03 years) | The Inventory of Parent and Peer Attachment Revised | Parent and peer attachment | Attachment, attitude to past, present, and future, sympathy, basic psychological needs | Variable-oriented study | Adolescents with secure attachment to parents had the most positive attitudes to past, present, and future. Adolescents with secure attachment to parents only or to both parents and peers showed the highest autonomy and competence. |
| Laible et al. (2000) | 89 adolescents (M_age_ 16.1 years) in a cross-sectional design | The Inventory of Parent and Peer Attachment Revised | Parent and peer attachment | Attachment, depression, anxiety, sympathy, aggression, math and English efficacy | Variable-oriented study | Adolescents with secure attachment to both parents and peers showed the best adjustment, and those low on both showed the lowest adjustment. Adolescents with secure peer but insecure parental attachment had better adjustment than those with secure parental but insecure peer adjustment. |
| Margolese et al. (2005) | 134 16–19-year-old adolescents in a cross-sectional study with questionnaires and a computer task with interpersonal vignettes | -Relationship Questionnaire | Mother, father, best friend, and romantic partner | Attachment, depression, cognitive attributions, ruminative coping, stress | Variable-oriented study | Insecure attachment with romantic partner was uniquely predictive of depression. |
| Markiewicz et al. (2006) | 682 Canadian students from three age categories: 12-15, 16-19 and 20-28 years in a cross-sectional design | Relationship Questionnaire  WHOTO (attachment functions) | Mother, father, best friend, romantic partner, oneself, other | Attachment and age | Variable-oriented study | Mothers were the most important attachment figures across ages, but for older adolescents, also romantic partners increased in importance. |
| Mayseless (2004) | 143 home-leaving Israeli male adolescents entering a 3-year mandatory military service in a longitudinal design | -Attachment Concerns Questionnaire  -WHOTO (attachment functions) | Parent and peer attachment | Attachment, inner resources of hardiness, adjustment to military | Variable-oriented study | Preference of peer attachment (instead of parents) was associated with better military adjustment. |
| McKay (2015) | 1724 12–16-year-old UK adolescents in a cross-sectional design | The Inventory of Parent and Peer Attachment Revised | Parent and peer attachment | Attachment, alcohol use, rules of alcohol use | Variable-oriented study | Insecure attachment to parents and secure attachment to peers were associated with problematic drinking. |
| Murphy et al. (2017) | 148 adolescents (M_age_ 15.68) in a cross-sectional design | The Inventory of Parent and Peer Attachment | Parent and peer attachment | Attachment and bullying | Variable-oriented study | Both secure parental and peer attachment were associated with less bullying, but secure attachment to peers without parental security also protected from bullying. |
| Nickerson & Nagle (2005) | 279 fourth, sixth, and eighth graders in a cross-sectional design | Attachment to parents and peers: People in My Life  WHOTO (attachment functions) | Parent and peer attachment | Attachment functions with parents and peers, age | Variable-based study | Parents retained secure base functions for all age groups, but older adolescents turned more to peers in other attachment functions. |
| Oldfield et al. (2016) | 203 English 11–16-yearolds in a cross-sectional design | nventory of Parent and Peer Attachment | Parent and peer attachment | Attachment, school connectedness, and mental health | Variable-oriented study | Insecure parental attachment was associated with conduct problems and emotional difficulties, whereas secure peer attachment and school connectedness were associated with prosocial behavior. |
| Oldfield et al. (2018) | 90 economically disadvantageous, 11–18year-old adolescents from Guatemala in a cross-sectional design | The Inventory of Parent and Peer Attachment | Parent and peer attachment | Attachment, school connectedness, and mental health | Variable-oriented study | Higher peer attachment security and lower school connectedness predicted more mental health difficulties. |
| Pitman & Scharfe (2010) | 302 undergraduate psychology students (M_age_ 20.08 years) in a cross-sectional design | -Relationship Scales Questionnaire  -Attachment Network Questionnaire | Mother, father, and close friends | Attachment, attachment network, distress | Variable-oriented study | Attachment anxiety was associated with distress in all adolescents. Attachment avoidance was associated with distress only among those with a predominantly family network, but not among those with a predominantly peer network. |
| Raja et al. (1992) | 935 15-year-old adolescents from a longitudinal cohort study in New Zealand | The Inventory of Parent and Peer Attachment | Parent and peer attachment | Attachment, mental health, life events, strengths | Variable-oriented study | Insecure attachment to parents was associated with lower mental health, and there was no compensation by secure attachment to peers. Those with secure attachment to both parents and peers reported the most strengths. |
| Rosenthal & Kobak (2010) | 212 high school and 198 college students in a cross-sectional design | The Important People Interview | Four most important people in one’s life and four additional peers | Attachment, behavior problems, parent-teen relationship | Variable-oriented study | With increasing age, romantic attachments ranked higher and fathers lower in the attachment hierarchy. Omitting the father and ranking friends high in the hierarchy were associated with more behavioral problems. |
| Sentino et al. (2018) | A review of eight studies examining parental attachment styles, sexual behaviors, and health outcomes in adolescent girls | Various measures | Attachment to parents | Parental attachment, adolescent sexual and health behaviors | Review study | Insecure parental attachment was associated with higher sexual risk in adolescent girls. |
| Umemura et al. (2018a) | 870 young Czech adults (M_age_ 21.57 years) in a three-wave longitudinal study | Experiences in Close Relationships- Relationship Structures  WHOTO (attachment functions) | Mother, father, best friend, romantic partner | Attachment and attachment functions | Variable-based study (multi-level modeling) | As romantic relationships progressed, they replaced friends, but not parents, as preferred attachment figures. |
| Umemura et al. (2018b) | 212 Czech 11–18-year-old adolescents in a cross-sectional design | Important People Interview (questionnaire) | Mother, father, another family member, a same-gender friend, an opposite-gender friend, romantic partner | Attachment, affective well-being, externalizing and internalizing problems | Variable-based study | Adolescents with multiple attachments had better adjustment, and adolescents who did not name their parents as attachment figures had lower adjustment. |
| Viejo et al. (2018) | 1025 Spanish 12–17- year-old secondary school students in a cross-sectional design | Important People Interview (questionnaire) | Father, mother, sibling, best friend, other friend, romantic partner | Attachment, age, and gender | Variable-based study | The bond with a romantic partner increases in importance in older adolescents. |
| Wang & Wang (2012) | 302 Chinese college students in a cross-sectional design | Relationship Questionnaire | Mother, father, best friend, romantic partner | Attachment, life satisfaction, self-esteem, relationship quality | Person- and variable-oriented study | Four hierarchical attachment profiles were found: all-average, all-secure, romantic-insecure, and peer-secure. The all-average profile had lower life satisfaction than the other profiles. |
| Wilkinson (2010) | 495 Australian 13–19-year-old students in a cross-sectional design | Adolescent Friendship Attachment Scale  Inventory of Parental and Peer Attachment | Attachment to parents and peers, and to best friend | Attachment, depression, self-esteem, school attitude, self-competence | Variable-oriented study | An adolescent’s relationship with the best friend has unique importance for well-being, beyond the effects of parental and general peer attachments. |
